# Interferon Kappa Is Important for Keratinocyte Host Defense against Herpes Simplex Virus-1

**DOI:** 10.1155/2020/5084682

**Published:** 2020-01-03

**Authors:** Yuanyuan Li, Yueqi Song, Leqing Zhu, Xiao Wang, Brittany Richers, Donald Y. M. Leung, Lianghua Bin

**Affiliations:** ^1^Biomedical Translational Research Institute, The First Affiliated Hospital, Jinan University, Guangzhou, Guangdong Province, China; ^2^Department of Pediatrics, National Jewish Health, Denver, Colorado, USA

## Abstract

Type I interferon kappa (IFN*κ*) is selectively expressed in human keratinocytes. Herpes simplex virus-1 (HSV-1) is a human pathogen that infects keratinocytes and causes lytic skin lesions. Whether IFN*κ* plays a role in keratinocyte host defense against HSV-1 has not been investigated. In this study, we found that *IFNκ* mRNA expression was induced by addition of recombinant IFN*κ* and poly (I:C); and its expression level was significantly greater than *IFNa2*, *IFNb1*, and *IFNL1* in both undifferentiated and differentiated normal human epidermal keratinocytes (NHEKs) under resting and stimulation conditions. Although IFNe was expressed at a relatively higher level than other IFNs in resting undifferentiated NHEK, its expression level did not change after stimulation with recombinant IFN*κ* and poly (I:C). HSV-1 infection inhibited gene expression of *IFNκ* and *IFNe* in NHEK. Silencing *IFNκ* in NHEK led to significantly enhanced HSV-1 replication in both undifferentiated and differentiated NHEK compared to scrambled siRNA-transfected cells, while the addition of recombinant IFN*κ* significantly reduced HSV-1 replication in NHEK. In addition, we found that IFN*κ* did not regulate protein expression of NHEK differentiation markers. Our results demonstrate that *IFNκ* is the dominant type of IFNs in keratinocytes and it has an important function for keratinocytes to combat HSV-1 infection.

## 1. Introduction

The interferon (*IFN*) *κ* gene was identified in 2001 [1]. It consists of 207 amino acids including a 27 amino acid signal peptide and has about 30% homology to other interferon genes. IFN*κ* was initially found to be constitutively expressed in human proliferating primary keratinocytes and could be induced significantly by IFN*β*, IFN-*γ*, and encephalomyocarditis virus (ECMV) [1]. Later, IFN*κ* mRNA was also found to be constitutively expressed in human innate immune cells including monocytes and dendritic cells [2]. Although IFN*κ* is expressed by limited cell sources, it activates the same signaling pathway as other type I IFNs by receptors of IFNRA1/IFNRA2 [1]. Because it is constitutively expressed in keratinocytes, IFN*κ* has been investigated for its role in human papillomavirus- (HPV-) involved human diseases. High-risk HPV were reported to inhibit IFN*κ* gene transcription in human cervical keratinocytes, and its expression is reduced and undetectable in HPV-positive human cervical keratinocytes [3–5].

Herpes simplex virus-1 (HSV-1) is a well-known human pathogen that establishes lifelong latency in the central nervous system [6, 7]. It triggers reactivation and lytic infections mainly in the skin and mucosal membrane, and these infections are often opportunistic and self-limited. However, under some conditions, such as immunodeficiency, and chronic usage of immune suppressants including steroids, some atopic dermatitis patients can develop severe forms of HSV-1 infections including eczema herpeticum and encephalitis [8–10].

In this study, we investigated the regulation of *IFNκ* and its function against HSV-1 in normal human epidermal keratinocytes (NHEKs). We found that *IFNκ* is the dominant type of IFNs compared to *IFNa2*, *IFNb1*, *IFN*e, and *IFNL1*; and it is critical for keratinocyte's host defense to control HSV-1 infections.

## 2. Methods and Materials

### 2.1. NHEK Cell Culture and Treatment

NHEKs were purchased from Thermo Fisher Scientific and maintained in EpiLife medium containing 0.06 mM CaCl_2_ and S7 supplemental reagent in 5% CO_2_ at 37°C. For NHEK differentiation, cells were cultured in EpiLife medium containing 1.3 mM CaCl_2_ for 2 days, then treated with recombinant human IFN*κ* (rhIFN*κ*), HSV-1, or PRR agonist poly (I:C) for additional 24 hours.

### 2.2. Virus Source, Cytokines, and PRR Agonist

HSV-1 (VR-733) was purchased from American Type Culture Collection (Manassas, VA). Recombinant human IFN*κ* was purchased from PBL Assay Science (Piscataway, NJ). Poly (I:C)-HMW/LyoVec™ and poly (I:C)-LMW/LyoVec™ were purchased from InvivoGen (San Diego, CA).

### 2.3. siRNA Knockdown Gene Expression

Three different *IFNκ* siRNA duplexes and control nontargeting scrambled siRNA duplexes were purchased from Life Technologies. The sequence for *IFNκ* siRNA #1 are as follows: sense: CCCUAUCCCUGGACUGUAAtt and antisense: UUACAGUCCAGGGAUAGGGtg; IFN*κ* siRNA #2 sense: GAUAGACAAUUUCCUGAAAtt and antisense: UUUCAGGAAAUUGUCUAUCct; IFN*κ* siRNA #3 sense: CACCUUCAAAUAUUGGAAAtt and antisense: UUUCCAAUAUU UGAAGGUGtg. NHEKs were plated in 24-well plates at 1 × 10^5^ per well the day before transfection. Cells were transfected with siRNA duplexes at a final concentration of 10 nM using lipofectamine 2000 according to the manufacturer's instructions (Invitrogen, Carlsbad, CA). After 24 hours of incubation, the cell culture medium was replaced with EpiLife supplemented either with 0.06 mM CaCl_2_ for 24 hours (undifferentiated condition, UD) or with 1.3 mM CaCl_2_ for 2 days (differentiated condition, D). HSV-1 at various multiplicity of infection (MOI) was then added to the cells for an additional 24 hours. After incubation with HSV-1, the cells were harvested for RNA extraction, qRT-PCR, and plaque assays.

### 2.4. Total RNA Extraction and qRT-PCR

Total RNA was extracted using RNeasy mini kit according to the manufacturer's guidelines (QIAGEN, MD). RNA was then reverse transcribed into cDNA using SuperScript® III reverse transcriptase from Invitrogen (Portland, OR) and analyzed by real-time PCR using an ABI Prism 7000 sequence detector (Applied Biosystems, Foster City, CA). Primers and probes for human *18S* (Hs99999901_s1), *IFNκ* (Hs00737883_m1), *IFNa2 (*Hs041892288_g1), *IFNb1* (Hs01077958_S1), *IFNL1* (Hs00601677_g1), and *IFNe* (Hs00703565_s1) were purchased from Applied Biosystems (Foster City, CA). The primers and probe of HSV-1 *gD* gene were described previously [11]. Quantities of all target genes in test samples were normalized to the corresponding *18S*.

### 2.5. Viral Plaque Assay

Vero cells were maintained in Minimum Essential Medium (MEM) with 5% of Fetal Bovine Serum (FBS). Cells were plated into 24-well dishes at 2 × 10^5^ to form monolayers. The following day, HSV-1-infected NHEK cell culture supernatants were frozen and thawed for three times to release the viral particles. The infectious media were then added to Vero cell monolayers with serial dilutions. After 2 hours of incubation, the infectious media were removed; and the cells were covered by 2% of methylcellulose made in MEM containing 2% FBS and cultured at standardized cell culture condition. Two days later, the viral plaque formation was visualized by 1% crystal violet staining.

### 2.6. Western Blot Protein Detection

Cells were lysed in 2x Laemmli sample buffer (Bio-Rad) and proteins were run on western blots. Antibodies against *β*-actin (clone W16197A) and KRT10 (clone DE-K10) were purchased from BioLegend; antibody against IVL (MA5-11803) was purchased from Thermo Scientific. Rabbit polyclonal anti-IFN*κ* (ab168119) was purchased from Abcam (Cambridge, MA).

### 2.7. Statistical Analysis

We used GraphPad prism software (version 5.03, San Diego, CA) for statistical analyses. Comparisons of expression levels were performed using ANOVA techniques and independent sample *t*-tests as appropriate. Differences were considered significant at *p* < 0.05.

## 3. Results

### 3.1. *IFNκ* Is the Dominant IFN Expressed in NHEK under Resting and Stimulated Conditions Compared to Other IFN Family Members

**Figure 1:**
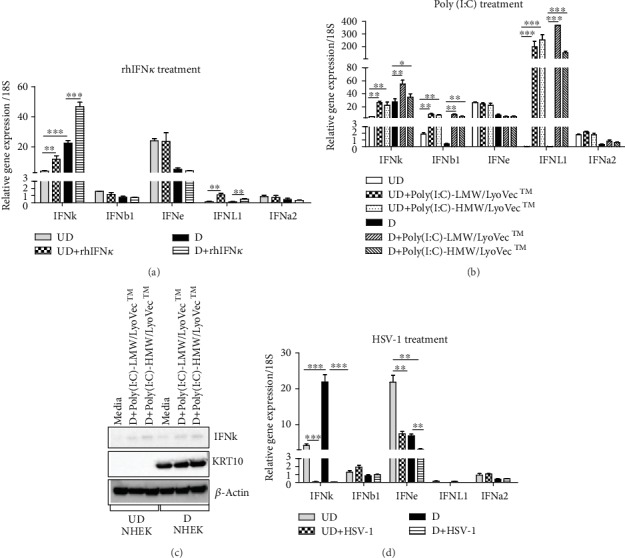
*IFNκ* is the dominant IFN in NHEK under resting and stimulated conditions compared to other IFN family members. Undifferentiated and differentiated NHEK cells were stimulated with rhIFN*κ* (10 ng/ml), poly (I:C) (1 *μ*g/ml), and HSV-1 (MOI of 0.05) for 24 hours. The cells were then harvested to evaluate *IFNa2*, *IFNb1*, *IFNκ*, *IFNe*, and *IFNL1* gene expression. (a) Gene expression was evaluated by real-time PCR in NHEK in the presence and absence of rhIFN*κ*. (b) Gene expression was evaluated by real-time PCR in NHEK in the presence and absence of poly (I:C). (c) Western blot assay to detect IFN*κ*, KRT10, and *β*-actin. (d) Gene expression was evaluated by real-time PCR in NHEK in the presence and absence of HSV-1 (MOI of 0.05). Data presented as mean ± SEM. One of three independent experiments is presented. ^∗^*p* < 0.05, ^∗∗^*p* < 0.01, and ^∗∗∗^*p* < 0.001.

To evaluate the relative importance of *IFNκ* in keratinocytes compared to other IFN family members, we investigated *IFNκ* expression levels in NHEK cells under both undifferentiated and differentiated conditions in the presence and absence of rhIFN*κ*, poly (I:C), and HSV-1. As shown in Figure 1(a), we found that *IFNκ* expression level was much greater than *IFNa2*, *IFNb1*, and *IFNL1*; and its expression was significantly induced by rhIFN*κ* in both undifferentiated (UD) and differentiated (D) NHEK; in addition, its expression level is significantly greater in differentiated NHEK than undifferentiated NHEK. *IFNa2* and *IFNb1* were not induced by rhIFN*κ*. Although *IFN*e mRNA was expressed at greater levels compared to other IFNs in undifferentiated NHEK, it was not upregulated further by rhIFN*κ*. *IFNL1* mRNA was extremely low in both undifferentiated and differentiated NHEK; interestingly, it was induced in the presence of rhIFN*κ*.

*IFNκ* has been reported to be upregulated in proliferating keratinocytes by poly (I:C) [4]. In this study, we investigated *IFNκ* gene expression in response to poly (I:C) stimulation in comparison with other IFN family members in undifferentiated and differentiated NHEK. Poly (I:C)-HMW/LyoVec™ and poly (I:C)-LMW/LyoVec™ contain different lengths of double-stranded RNA which activate RIG-1-like receptor-mediated signaling pathways [12]. As shown in Figure 1(b), three IFNs, *IFNκ*, *IFNb1*, and *IFNL*1, were significantly induced by poly (I:C)-LMW/LyoVec™ and poly (I:C)-HMW/LyoVec™ (1 *μ*g/ml) in both undifferentiated and differentiated NHEK, while *IFNa2* and *IFNe* had no change. We were able to detect IFN*κ* protein in cell lysates collected from poly (I:C)-stimulated NHEK in both undifferentiated and differentiated cells (Figure 1(c)). IFN*κ* protein was not detected in media alone-treated undifferentiated NHEK by western blot assay, but it was detectable in media alone-treated differentiated NHEK. These data demonstrate that IFN*κ* is significantly upregulated in differentiated NHEK.

We also investigated how HSV-1 infection affects *IFNκ* gene expression in NHEK. As shown in Figure 1(d), HSV-1 infection inhibited *IFNκ* and *IFNe* expression in undifferentiated and differentiated NHEK, but *IFNb1*, *IFNL1*, and *IFNa2* were not affected.

### 3.2. Silencing IFN*κ* Expression Leads to Enhanced HSV-1 Replication in NHEK

**Figure 2:**
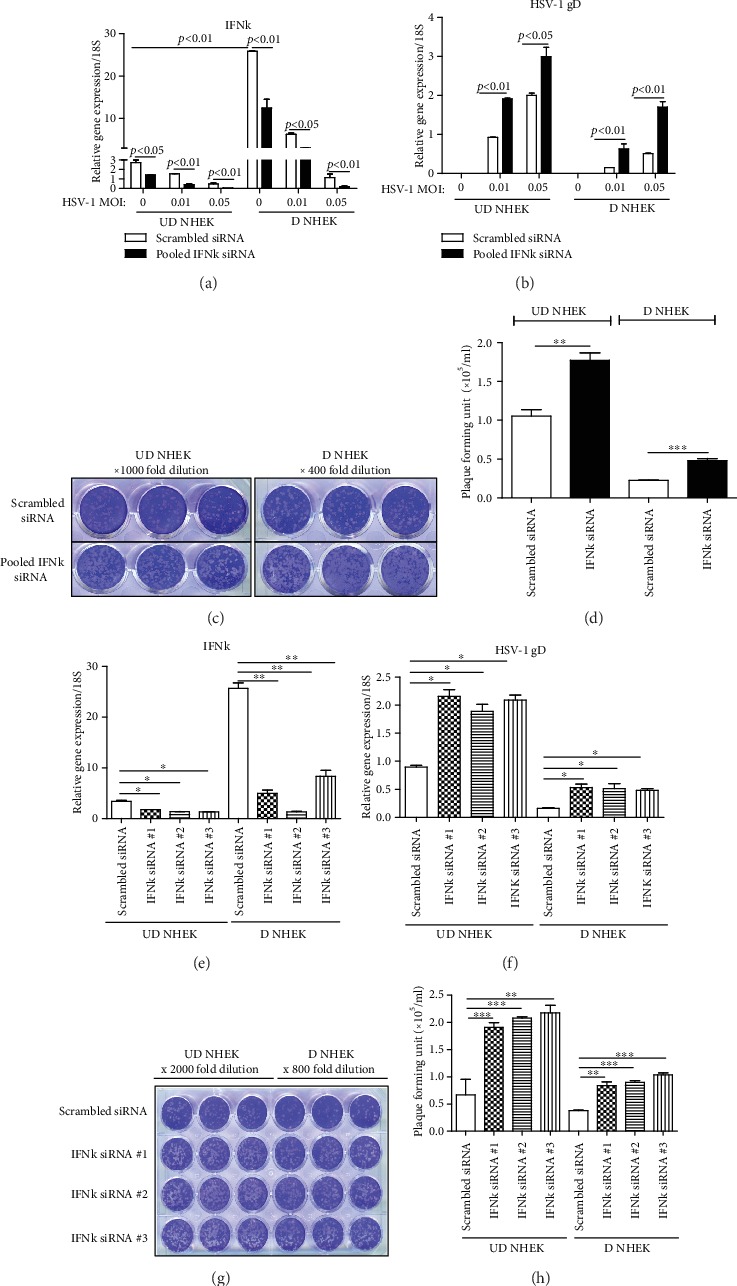
Silencing IFN*κ* leads to enhanced HSV-1 replication in NHEK. Undifferentiated (UD) and differentiated (D) NHEK cells were transfected with scrambled siRNA and IFN*κ* siRNA duplexes. The cells were then infected with HSV-1 for an additional 24 hours before harvested. (a) *IFNκ* transcripts and (b) HSV-1 *gD* transcripts were evaluated by real-time qRT-PCR. (c) Representative viral plaque assay results were shown. (d) Quantitative results of HSV-1 plaque assay using infectious materials collected from NHEK infected with HSV-1 (MOI of 0.05). (e) *IFNκ* transcripts and (f) HSV-1 *gD* transcripts were evaluated by real-time qRT-PCR. (g) Representative viral plaque assay results were shown. (h) Quantitative results of HSV-1 plaque assay using infectious materials collected from NHEK HSV-1 (MOI of 0.05). Data is presented as mean ± SEM. One of four independent experiments is presented. ^∗^*p* < 0.05; ^∗∗^*p* < 0.01.

Although IFN*κ* was found to protect host cells from ECMV and HCV infections [13], it has not been investigated whether IFN*κ* could protect keratinocytes from HSV-1 infection. To test IFN*κ* function in keratinocytes against HSV-1 infection, we silenced IFN*κ* gene expression in NHEK in undifferentiated and differentiated NHEK and then evaluated HSV-1 replication in *IFNκ*-silenced NHEK. HSV-1 replication in NHEK cells was evaluated by real-time qRT-PCR of HSV-1 *gD* gene and viral plaque assays. Using a pool of siRNA duplexes to inhibit *IFNκ* gene expression in NHEK and cells transfected with scrambled siRNA as controls, we found that *IFNκ* gene expression was sufficiently inhibited by siRNA silencing in both undifferentiated and differentiated NHEK cells (Figure 2(a)). HSV-1 *gD* expression was significantly increased in *IFNκ-*silenced cells compared to scrambled siRNA-treated cells (Figure 2(b)). We further performed viral plaque assays and confirmed that IFN*κ*-silenced NHEK produced increased HSV-1 plaques than controls (Figures 2(c) and 2(d)). To confirm these results were not an off-target effect, we used three different siRNA duplexes to target *IFNκ* in undifferentiated and differentiated NHEK cells. As shown in Figure 2(e), three *IFNκ* siRNA duplexes targeting different regions of *IFNκ* gene efficiently inhibited *IFNκ* gene expression. HSV-1 *gD* gene expression was significantly increased in *IFNκ-*silenced NHEK cells (Figure 2(f)). We further used viral plaque assays to evaluate the production of viral infectious particles. As shown in Figures 2(g) and 2(h), *IFNκ-*silenced NHEK produced significantly increased viral plaques compared to the control cells. These results demonstrated that IFN*κ*-silenced NHEKs are more susceptible to HSV-1 infection.

### 3.3. Addition of rhIFN*κ* Inhibits HSV-1 Replication in NHEK

**Figure 3:**
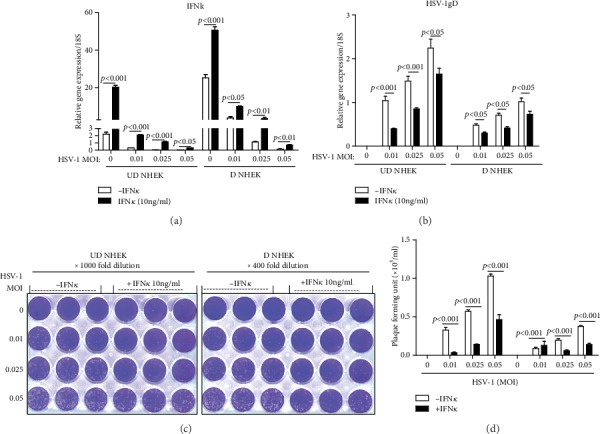
Addition of rhIFN*κ* inhibits HSV-1 replication in NHEK. Undifferentiated (UD) and differentiated (D) NHEK cells were treated with rhIFN*κ* (10 ng/ml) and indicated MOI of HSV-1 for 24 hours. Cells were then harvested for real-time qRT-PCR. (a) *IFNκ* and (b) HSV-1 *gD* transcripts were evaluated by real-time qRT-PCR. (c) Representative viral plaque assay results were shown. (d) Quantitative results of HSV-1 plaque assays. Data presented as mean ± SEM. One of three independent experiments is presented.

Since silencing IFN*κ* leads to increased HSV-1 infection, we investigated whether the addition of rhIFN*κ* to NHEK cells could reduce HSV-1 replication in these cells. We found that HSV-1 significantly inhibited *IFNκ* gene expression, but the addition of rhIFN*κ* could increase the endogenous *IFNκ* gene expression compared to control treatments (Figure 3(a)). HSV-1 *gD* gene expression was significantly reduced in NHEK cells in the presence of rhIFN*κ* compared to the absence of rhIFN*κ* (Figure 3(b)); NHEK with rhIFN*κ* treatment resulted in significantly reduced viral plaques compared to cells without rhIFN*κ* treatment (Figures 3(c) and 3(d)). These data demonstrate that IFN*κ* is capable of inhibiting HSV-1 replication in NHEK cells.

### 3.4. IFN*κ* Does Not Regulate NHEK Differentiation

**Figure 4:**
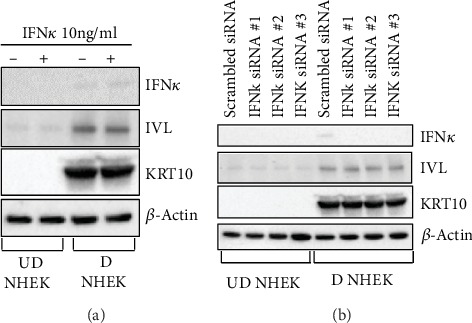
IFN*κ* does not regulate NHEK differentiation. (a) Western blot assay to detect IFN*κ*, IVL, KRT10, and *β*-actin in undifferentiated (UD) and differentiated (D) NHEK in the presence and absence of rhIFN*κ*. (b) Western blot assay to detect IFN*κ*, IVL, KRT10, and *β*-actin in IFN*κ*-silenced undifferentiated (UD) and differentiated (D) NHEK. One of three independent experiments is presented.

As shown in Figures 2 and 3, we found that undifferentiated NHEK supports more HSV-1 replication than differentiated NHEK; therefore, we investigated whether IFN*κ* regulates NHEK differentiation program and consequently affects HSV-1 replication by altering NHEK differentiation status. Keratin 10 (KRT10) is a marker of spinous layer of the epidermis, and involucrin (IVL) is a marker of granular and stratum corneum layers [14]. We found that the addition of IFN*κ* and silencing *IFNκ* did not change the expression of KRT10 and IVL in NHEK (Figures 4(a) and 4(b)). These data suggest that IFN*κ* does not regulate NHEK differentiation; thus, the mechanism by which it inhibits HSV-1 infection is not by regulating NHEK differentiation.

## 4. Discussion

Type I IFNs comprise more than 20 homologous cytokines that were discovered based on their antiviral activities [15]. All type I IFNs including IFN*κ* use a common type I IFN receptor complex that comprises two chains of IFNAR1 and IFNAR2. Upon ligand binding, IFNAR1 and IFNAR2 dimerize and initiate a signaling cascade that includes phosphorylation of Tyk2 and Jak1 tyrosine kinases and subsequent phosphorylation of the STAT1 and STAT2 proteins. Association of the phosphorylated STAT proteins with IRF9 forms the interferon-stimulated gene factors 3 multi-subunit complex, which translocates to the nucleus and binds to interferon-stimulated response elements in the upstream of IFN-inducible genes, and subsequently activates hundreds of genes to confer antiviral, antitumor, and immune modulatory activities [16, 17]. The type I IFN cytokines have shown differences in their cell sources, receptor affinities, and gene targets as well as biological activities [17]. In order to define the importance of IFN*κ* in keratinocyte innate immune responses, we compared *IFNκ* mRNA expression levels with four other IFNs (*IFNa2*, *IFNb1*, *IFNL1*, and *IFNe*) under both resting and stimulation conditions in both undifferentiated and differentiated NHEK (Figure 1). The rationale for us to choose these four IFNs are as follows. (1) *IFNa2/IFNb1* are the most studied type I IFNs and IFNa2 has been used in clinical treatment of hepatitis and skin malignancies for decades [18, 19]; IFN*β* is also used for multiple sclerosis treatment [20]. (2) IFNL1 is the representative cytokine of IFN-*λ* family, an emerging master regulator of innate and adaptive immune systems for mucosal membrane tissues [21]. (3) IFN*ε* has been reported to protect female reproductive tracts from viral and bacterial infections [22]. Our data for the first time reveals that IFN family members respond differently to the same stimulation in keratinocytes, and IFN*κ* is the dominant type of IFNs in keratinocytes under unstimulated and stimulated conditions of itself, poly (I:C), and HSV-1 in both undifferentiated and differentiated conditions, suggesting that IFN*κ* may be the dominant IFN of skin host defense against viral infections. Additionally, we found that IFN*κ* gene expression was induced by the addition of rhIFN*κ*, suggesting that this gene can be regulated by the forward feedback regulation mechanism in keratinocyte.

The importance of type I IFN in HSV-1 resistance has been demonstrated by studies using type I IFN receptor knockout mice. Mice lacking type I IFN signaling have significantly decreased survival after ocular and footpad inoculation of HSV-1 [23, 24]. In addition, human patients suffering from herpes simplex encephalitis often have defects in type I IFN signaling [25–27]. On the other hand, previous studies have found HSV-1 has developed multiple mechanisms to dampen type I interferon production in different types of cells to facilitate infection [28]. For example, HSV-1 US3, a tegument protein kinase, can reduce TLR3 gene expression thus inhibiting TLR3-mediated type I IFN response [29]; HSV-1 US11, an RNA-binding tegument protein, can interact with RIG-1 and MDA5 and prevent these proteins from interacting with the downstream adaptor protein, MAVS, and consequently inhibit IFN*β* production [30, 31]. In this study, we found the gene expression of *IFNκ* was significantly inhibited by HSV-1 in both undifferentiated and differentiated NHEK in a dose-dependent manner (Figure 2(a)), suggesting that HSV-1 has strong antagonistic effects against IFN*κ* in keratinocytes. Thus, from the perspective of HSV-1-invading strategy, we speculate that IFN*κ* is one of the critical targets for the virus to overcome in order to establish effective infection in keratinocytes. Indeed, we found that HSV-1 replication was significantly enhanced in IFN*κ*-silenced NHEK cells compared to control cells; and treatment of exogenous rhIFN*κ* significantly restrained HSV-1 replication in NHEK. These results demonstrate that IFN*κ* is important for keratinocyte innate immunity against HSV-1 infection and IFN*κ* may be an effective therapeutic target for HSV-1 skin infections.

In this study, we found that differentiated keratinocytes were more resistant to HSV-1 infection compared to undifferentiated cells (Figures 2 and 3). Interestingly, we found IFN*κ* mRNA and protein were significantly increased in differentiated NHEK. These data suggest that increased IFN*κ* gene expression in differentiated keratinocytes may be one of the mechanisms by which differentiated NHEK has increased resistance to HSV-1 infection.

In summary, our data in this study demonstrate that IFN*κ* is the dominant type of IFNs in human keratinocytes and it is important for human keratinocytes to control HSV-1 infection.

## Data Availability

All of the data used to support the findings of this study are included within the article.

## Abbreviations

HSV: Herpes simplex virus
IFN: Interferon
ISG: IFN-stimulated gene
IVL: Involucrin
KRT10: Keratin 10
MOI: Multiplicity of infection
NHEKs: Normal human epidermal keratinocytes
PRR: Pathogen recognition receptor
rhIFN*κ*: Recombinant human IFN-*κ*
qRT-PCR: Quantitative reverse transcription polymerase chain reaction
siRNA: Small interfering RNA.
